# Improved non-singular fast terminal sliding mode PMSM control strategy

**DOI:** 10.1371/journal.pone.0328004

**Published:** 2025-07-11

**Authors:** Jingbin Yan, Haoxuan Hu

**Affiliations:** School of Electrical and Electronic Engineering, Harbin University of Science and Technology, Harbin, China; University of Botswana Faculty of Engineering and Technology, BOTSWANA

## Abstract

To strengthen the overall control capability of the permanent magnet synchronous motor (PMSM) system, an improved non-singular fast terminal sliding-mode control strategy is proposed, that can simultaneously improve the shortcomings of traditional PI and sliding mode control (SMC), which include a large overshoot, large jitter, and poor robustness. First, a new type of non-singular fast terminal sliding-mode surface was constructed according to a surface-mounted PMSM, which avoids singular phenomena in the system. An improved power-reaching law was designed, which not only enables the control system to quickly approach the error to zero, but can also better suppress the chattering phenomenon. Moreover, an adaptive law is introduced to regulate the reaching law coefficient in real time, which further increases the control precision. The system stability was proven using the Lyapunov stability theory. Second, a beat-free predictive current controller was designed for the current loop to further strengthen the system's dynamic response. The matching disturbance of the extended state observer (ESO) is subsequently introduced, and the observed value is transmitted to the designed speed controller in real time. The advantages of the proposed strategy were analyzed through simulations, and its reliability was verified experimentally. Finally, through the simulation and experimental results, it was concluded that the improved non-singular fast terminal sliding mode control (INFTSMC) strategy for PMSM systems can overcome the shortcomings of traditional PI and sliding-mode control systems and increase the response speed and anti-interference ability of the system.

## 1. Introduction

The permanent magnet synchronous motor (PMSM) is a complex object with strong coupling, multiple variables, and non-linear and variable parameters. To achieve better control performance, it is necessary to adopt certain control algorithms [1]. When the motor internal part changes or the motor control system is affected by external disturbances, traditional PI control cannot meet the control requirements. Accordingly, a PI fuzzy controller was designed in [2]. However, $k_{p}$ and $k_{i}$ fuzzy control rules need to be designed simultaneously, which increases the system complexity.

To strengthen the tracking accuracy and maintain stability, various control strategies have been proposed [3–5], such as sliding-mode control (SMC), active disturbance rejection control, and model predictive control. Sliding mode variable structure control is extensively utilized because of its notable benefits including swift responsiveness, resilience to parameter variations, and straightforward physical application [6]. However, traditional SMC adopts a linear sliding-mode surface, which ensures that the system converges to the equilibrium state when the system time tends to infinity [7–10]. System state variables were introduced into the new reaching law to make the reaching speed dependent on the system state and simultaneously ensure that the system state reaches the sliding-mode surface and converges to zero in finite time in [11]. A finite-time tracking-control strategy was proposed in [12] for a quadrotor system affected by external interference and model uncertainties. This policy provides preassigned performance guarantees. In [13–15], the adaptive rules of the sliding-mode parameters were redesigned to enhance the system's anti-interference ability. However, the traditional integrated sliding-mode surface was adopted, resulting in a slow convergence speed and large electromagnetic torque jitter after stable motor operation In [16], these problems were addressed by using terminal SMC (TSMC). However, when the system variable $x_{1}$ approaches to 0 and $x_{2}$ is not equal to 0, the control input appears infinity, that is singularity. Some scholars have proposed non-singular fast TSMC strategies to address these issues [17,18]. Moreover, a non-singular fast TSMC strategy was adopted in [19,20] to avoid the singularity phenomenon in the algorithm and restrain the problems caused by other factors, and it also guarantees that the system's tracking error converges to zero within a finite time, weakening jitter and enhancing system robustness. When the current sensor fails, the measured current becomes inaccurate, resulting in the feedback control law being ineffective in design. Reference [21] introduced a sensorless continuous SMC strategy to address these issues. The flutter effect stemming from discontinuous SMC switching can be alleviated using a novel boundary-based saturation function. However, its control is complicated. In [22] and [23], a hyperbolic sine function was used to design the approach law. They constructed a variable cutoff low-pass filter (LPF) two-stage structure and modified the back electromotive force observer to suppress high-frequency components, which has good adjustment performance. However, it increases the system complexity and reduces its stability. A load-adaptive disturbance observer was designed in [24] to track parameter changes and load disturbances effectively, and the control effect was slightly improved. In [25], a novel nonlinear sliding-surface function with a double-closed-loop voltage and current structure was designed. This strategy combines two features: TSMC and fractional order computing (FOC). The adaptive fractional order TSMC(AFTSMC) showed insignificant enhancement in accelerating the output voltage response during load variations. Nonetheless, it attains a quicker dynamic response, a reduced steady-state error rate, and lower overshoot, with increased complexity in control. In [26–29], a new approach law was designed that can simultaneously strengthen the system velocity response and weaken system jitter. An extended observer was additionally developed to monitor external disturbances in real-time, thus enhancing the dynamic and steady-state characteristics of the position-tracking system. However, this strategy is easily affected by parameters, and the control algorithm is complicated. To strengthen the quality of the approach, the authors of [30] optimized its performance using a novel SMC strategy that is based on the approximation law. An adaptive gain-based advanced SMC reaching law (ASMCRL) was introduced to accelerate the convergence of the system state to the sliding-mode surface. In [31], the ASMC algorithm was combined with SMC to achieve accurate PMSM control and improve its performance. However, further simplification of the algorithm is necessary to minimize the impact of parameters on its control effect.

In this paper, a new type of non-singular fast terminal sliding mode surface is constructed to avoid the singular phenomenon in the system and further enhance the control precision. The introduction of beat free current predictive control obtain better dynamic response performance. Furthermore, an enhanced expansion observer is introduced for real-time observation of load disturbances, providing feedback to the speed controller.

The key contributions of this paper are highlighted as follows:

1. To continuously enhance the control performance of PMSM system, an improved non-singular fast terminal sliding mode control (INFTSMC) strategy is used for the speed loop, and a new power reaching law is designed which can make the control system quickly converge to zero and reduce jitter.
2. The adaptive law is introduced to regulate the coefficient of the reaching law in real time to further improve the control accuracy.
3. For the current loop, the introduction of beat free current predictive control strategy obtain excellent dynamic response performance. The high-gain extended observer is introduced to accurately observe the load disturbance in real time, and compensate it in the speed controller, which greatly improves the anti-interference ability of the system.

The remainder of this paper is organized as follows. In the second section, a mathematical model is established for the surface-mounted PMSM. Firstly, the improved non-singular fast terminal sliding-mode controller is designed in the speed loop. Secondly, a beat free current prediction controller is designed for the current loop, and the ESO is introduced to feed the load disturbance to the speed controller in real time. In the third section, software simulations are used to analyze the advantages of the designed control strategy. In the fourth section, the system reliability is verified experimentally. Finally, Section 5 concludes the paper.

## 2. PMSM mathematical model

To construct the speed controller and overall simulation model reasonably and conveniently, the surface-attached PMSM (SPMSM) is generally considered as an example.

The mathematical SPMSM model in d-q axes rotational reference frame is written as follows:


$$\begin{array}{c} \left\{ \begin{array}{c} u_{d}=Ri_{d}+L_{d}\frac{di_{d}}{dt}-L_{q}\omega_{e}i_{q}\\ u_{q}=Ri_{q}+L_{q}\frac{di_{q}}{dt}+\omega_{e}L_{q}i_{d}+\omega_{e}\varphi_{f} \end{array}\right. \end{array}$$ (1)

The electromagnetic torque equation is expressed as


$$\begin{array}{c} T_{e}=\frac{3}{2}p_{n}i_{q}\left[i_{d}\left(L_{d}-L_{q}\right)+\varphi_{f}\right] \end{array}$$ (2)

The mechanical motion equation is as follows:


$$\begin{array}{c} J\frac{d\omega_{m}}{dt}=T_{e}-T_{L}-B\omega_{m} \end{array}$$ (3)

Where, R is the stator winding phase resistance; $u_{d}$ and $u_{q}$ are the d-q axial voltages, respectively; $i_{d}$ and $i_{q}$ are the d-q axial currents, respectively; $p_{n}$ is the motor pole number; $\omega_{e}$ is the electric angular velocity; $\omega_{m}$ is the mechanical angular velocity; $L_{d}$ and $L_{q}$ are the d-q axial inductance components of the motor, respectively; $\varphi_{f}$ is the motor flux; $T_{L}$ is the load torque; $T_{e}$ is the electromagnetic torque; J is the inertia coefficient; B is the friction coefficient. For the SPMSM, $L_{d}=L_{q}$. Therefore, the electromagnetic torque equation can be changed to


$$\begin{array}{c} T_{e}=\frac{3}{2}p_{n}i_{q}\varphi_{f} \end{array}$$ (4)

If the $i_{d}=0$ control scheme is used, Eq (4) is substituted into Eq (3) to obtain


$$\begin{array}{c} \frac{d\omega_{m}}{dt}=\frac{3p_{n}\varphi_{f}}{2J}i_{q}-\frac{T_{L}}{J}-\frac{B}{J}\omega_{m} \end{array}$$ (5)

Analysis and design of speed controller

SMC is a nonlinear control strategy based on designing a sliding surface (s(x)=0 to drive the system state to converge in finite time and subsequently maintain stable sliding motion. The motion state of the SMC system consists of the initial sliding mode moving along the switching direction towards the equilibrium point and the approaching mode moving through the sliding mode under continuous control, which determines the motion quality under the approaching state. However, traditional sliding-mode controllers have a significant disadvantage. When the system reaches the sliding-mode surface, the system's state trajectory does not follow along it. Instead, it oscillates up and down. This is referred to as the SMC jitter phenomenon. This is because the state variable motion of a SMC system typically consists of two parts: the sliding phase and the reaching phase. The primary role of the control law is to guarantee that the system's state trajectory attains the intended sliding surface and converges in the reaching phase. When the system diverges from the sliding surface, selecting an apt control law allows the SMC system's state trajectory to revert to it more swiftly. Upon reaching the sliding surface, the intended arrival speed diminishes to zero, sustaining the sliding state theorem. However, due to sliding inertia, maintaining zero speed on the sliding surface is challenging. Hence, the sliding state oscillates along the sliding surface's sides.

The traditional reaching law generally selects the isokinetic, exponential, and power reaching laws. Although SMC has certain advantages, it also has disadvantages and limitations. The first is the jitter problem. The traditional approach produces high-frequency oscillations near the sliding-mode surface, according to Lyapunov stability theory, leading to chattering. This type of jitter can negatively affect the system performance and lifetime, which is unacceptable in certain applications. Another limitation is that its control signal is too large. Owing to the saturation function used in the approach law, the control signal may produce a large amplitude near the sliding-mode surface, which may lead to saturation or actuator over-response, affecting the system stability and reliability. The sliding surface is a hyper-surface that determines the desired dynamic behavior of the system. Once the system state reaches the sliding surface, its motion is governed by the dynamics defined by the surface, becoming insensitive to the original system parameters or external perturbations. The selection of the sliding surface typically depends on the system order. To ensure stability, the system dynamics must exhibit exponential convergence on the sliding surface. Moreover, physical constraints on the control input must be considered. An excessively large slope of the sliding surface may lead to overly aggressive control inputs, resulting in severe chattering.

**Fig 1:**
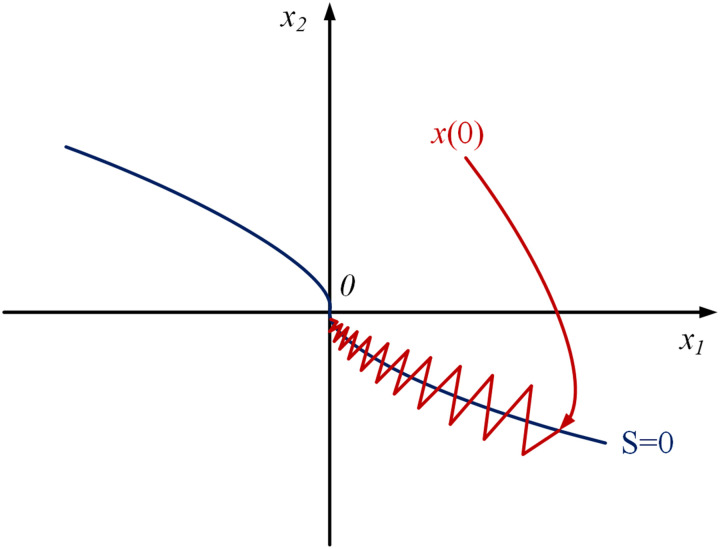
Phase trajectory of terminal sliding mode control.

By utilizing a non-linear sliding surface function, the TSMC approach guarantees finite-time convergence of the system state to the equilibrium point, addressing the limitations of SMC. The TSMC phase trajectory is shown in Fig 1.

$x_{1}$, $x_{2}$ are the system state variables, with $\dot{x_{1}}=x_{2}$.

The terminal sliding surface function is designed as:


$$\begin{array}{c} s=x_{2}+\beta x_{1}^{\frac{m}{n}} \end{array}$$ (6)

Where, m and n are positive odd numbers and 0<m/n<1, β is a positive constant.

Let s=0, then


$$\begin{array}{c} x_{2}=\frac{dx_{1}}{dt}=-\beta x_{1}^{\frac{m}{n}} \end{array}$$ (7)

Then, the derivative of time is


$$\begin{array}{c} dt=-\frac{1}{\beta }x_{1}^{-\frac{m}{n}}dx_{1} \end{array}$$ (8)

By integrating both ends of Eq (8), the convergence time from any initial state to the equilibrium state of the system can be established as


$$\begin{array}{c} t=\frac{n}{\beta (n-m)}{\left|x_{1}(0)\right|}^{\frac{n-m}{n}} \end{array}$$ (9)

As there will be a negative exponential power term about $x_{1}$ in the control law, the control input tends to infinity when $x_{1}\to 0$ and $x_{2}\neq 0$, a singularity. To ensure the finiteness of the control input, it is necessary to design a non-singular TSMC (NTSMC) strategy to avoid this strange phenomenon.

Therefore, the non-singular terminal sliding surface function is designed as


$$\begin{array}{c} s_{N}=x_{2}+kx_{1}+\alpha {\left|x_{1}\right|}^{\frac{n}{m}} \end{array}$$ (10)

Where, $k\in R^{+}$, $\alpha \in R^{+}$, *m* and *n* are positive odd numbers, $\frac{1<n}{m}<2$, which are parameters set in the sliding surface. This non-singular terminal sliding surface design considers the advantages of linear and non-linear sliding surfaces. The first two terms of Eq (10) are the conventional linear sliding surface forms, which play a major role when the distance is relatively long, and the latter term accelerates the convergence rate when it is close to the equilibrium point. Therefore, the integration of the two factors can guarantee global convergence of the system state throughout the entire convergence process.

Let $s_{N}=0$, then


$$\begin{array}{c} \dot{x_{1}}=-kx_{1}-\alpha {\left|x_{1}\right|}^{\frac{n}{m}} \end{array}$$ (11)

By integrating both ends of the Eq (11), the convergence time of the system state is


$$\begin{array}{c} t=\frac{n}{k(n-m)}[\ln(k\;{\left|x_{1}(0)\right|}^{\frac{n-m}{n}}+\alpha )-ln\alpha ] \end{array}$$ (12)

Comparing Eq (9) and Eq (12), the convergence time can be changed by changing the values of *α*, *m*, and *n*, so that the sliding variable converges to zero more quickly.

Taking the first derivative of Eq (10) with respect to time, we obtain


$$\begin{array}{c} \dot{s_{N}}=\dot{x_{2}}+kx_{2}+\alpha \frac{n}{m}{\left|x_{1}\right|}^{\frac{n}{m}-1}x_{2} \end{array}$$ (13)

From the Eq (13), we can see that when $\left(\frac{n}{m}\right)-1>0$, infinity does not occur when $x_{1}\to 0$. Therefore, the system is non-singular.

Defining the PMSM system velocity error state as


$$\begin{array}{c} x_{1}=\omega_{m}^{*}-\omega_{m} \end{array}$$ (14)

$$\begin{array}{c} x_{2}=\dot{x_{1}}=-{\dot{\omega }}_{m} \end{array}$$ (15)

Where, $\omega_{m}^{*}$ is the desired angular velocity of the motor and $\omega_{m}$ is the given and actual mechanical angular velocities, respectively.

By combining Eqs (3), (4) and (15), we have


$$\begin{array}{c} x_{2}=-{\dot{\omega }}_{m}=-\frac{3p_{n}\varphi_{f}}{2J}i_{q}+\frac{T_{L}}{J}+\frac{B}{J}\omega_{m} \end{array}$$ (16)

A new non-singular fast terminal sliding-mode controller is designed. The sliding-mode surface function is defined as follows:


$$\begin{array}{c} s=x_{1}(t)+p\int_{0}^{t}x_{1}(\tau )d\tau +q\int_{0}^{t}{\left|x_{1}(\tau )\right|}^{\lambda }sgn\left(x_{1}(\tau )\right)d\tau \end{array}$$ (17)

Where, $x_{1}$ is the system state error, p>0, q>0, and 0<λ<1.

Differentiating Eq (17) yields


$$\begin{array}{c} \dot{s}=x_{2}+px_{1}+q{\left|x_{1}\right|}^{\lambda }sgn\left(x_{1}\right) \end{array}$$ (18)

Substituting Eq (16) into (18), we obtain


$$\begin{array}{c} \dot{s}=-\frac{3p_{n}\varphi_{f}}{2J}i_{q}+\frac{T_{L}}{J}+\frac{B}{J}\omega_{m}+px_{1}+q{\left|x_{1}\right|}^{\lambda }sgn\left(x_{1}\right) \end{array}$$ (19)

Let $\frac{3p_{n}\varphi_{f}}{2J}=D$, $i_{q}=u$, and adopting the traditional approach rate, then the SMC law of the speed controller is


$$\begin{array}{c} u=\frac{1}{D}\left[\frac{T_{L}}{J}+px_{1}+q{\left|x_{1}\right|}^{\lambda }sgn\left(x_{1}\right)+\frac{B}{J}\omega_{m}+ksgn(s)\right] \end{array}$$ (20)

Where, k denotes the SMC switching gain. It can be concluded from Eq (20) that, because of the existence of the discontinuous term ksgn(s in the vacancy rate, the system produces buffering. Additionally, when k is larger, the system robustness is stronger, and the approach speed is faster. However, the jitter also increases. To realize a stable system operation, an improved power-reaching rate and the method of utilizing the saturation function replacing the sign function are designed to suppress the inherent chattering phenomenon of the sliding mode.

The improved power reaching rate is designed as follows:


$$\begin{array}{c} \left\{ \begin{array}{c} \dot{s}=-cs-|s{|}^{s\omega }f(s)\\ s\omega =\frac{1+|s|}{|s|+\in }\\ f(s)=1-\frac{2}{1+e^{hs}} \end{array}\right. \end{array}$$ (21)

**Fig 2:**
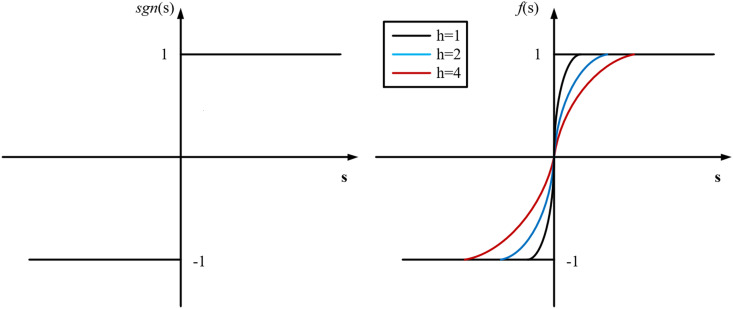
Curve of the function f(s and function. sgn(s).

Where,cis a constant greater than zero. ∈ is a small positive number, which can avoid the singularity when |s|=0. Using the function f(s to replace the sign function sgn(s can make the system approximation in sliding mode simpler and smoother. The curves of the f(s and sgn(s functions are shown in Fig 2. For comprehensive consideration, h=4 makes it smoother.

To satisfy the high-precision control requirements of a PMSM system, an adaptive law was introduced to adjust the parameters of the improved reaching law in real time.

Let


$$\begin{array}{c} c=c\cdot \frac{\left|x_{1}\right|}{1+\left|x_{1}\right|} \end{array}$$ (22)

Where, c affects the convergence rate. In the convergence phase, the greater the c value, the faster the convergence. At this time, $\left|x_{1}\right|$ is larger, $\left|x_{1}\right|/\left(1+\left|x_{1}\right|\right)\approx 1$. When the sliding mode reaches this stage, the smallercis, the smaller the jitter will be. In this case, $\left|x_{1}\right|$ is smaller, $\left|x_{1}\right|/\left(1+\left|x_{1}\right|\right)\ll 1$.

From Eq (11), the SMC law of the speed controller is obtained as follows:


$$\begin{array}{c} u=\frac{1}{D}\left[\frac{T_{L}}{J}+px_{1}+q{\left|x_{1}\right|}^{\lambda }sgn\left(x_{1}\right)+\frac{B}{J}\omega_{m}+c\cdot \frac{\left|x_{1}\right|}{1+\left|x_{1}\right|}s+|s{|}^{s\omega }f(s)\right] \end{array}$$ (23)

The Lyapunov stability principle was used to verify the designed controller's stability, and the Lyapunov function $V=\frac{1}{2}s^{2}$was selected


$$\begin{array}{c} \dot{V}=s\dot{s}=s\left[x_{2}+px_{1}+q{\left|x_{1}\right|}^{\lambda }sgn\left(x_{1}\right)\right]\\ =-s\left[c\cdot \frac{\left|x_{1}\right|}{1+\left|x_{1}\right|}s+|s{|}^{s\omega }f(s)\right]\ll 0 \end{array}$$ (24)

As indicated in Eq (24), the designed control system is stable.

### Design of high gain extended observer

According to Eq (3), and considering the load torque and the change in system parameters, the following can be obtained:


$$\begin{array}{c} {\dot{\omega }}_{m}=-\left(\frac{B}{J}+\Delta a\right)\omega +\left(\frac{3p_{n}\phi_{f}}{2J}-\Delta b\right)-\left(\frac{T_{L}}{J}+\Delta c\right)=a\omega +bu-d \end{array}$$ (25)

Where, $a=-\frac{B}{J}$, $b=\frac{3p_{n}\phi_{f}}{2J}$, and u is the control law to be designed. Δa,Δb and Δc represent the motor parameter changes. Finally, d indicates that the system's overall disturbance encompasses external load disturbances and internal parameter perturbations.

According to Eq (23) and Eq (25), the designed control rate is


$$\begin{array}{c} u=\frac{1}{D}\left[\frac{d}{J}+px_{1}+q{\left|x_{1}\right|}^{\lambda }sgn\left(x_{1}\right)+\frac{B}{J}\omega_{m}+cs+|s{|}^{s\omega }f(s)\right] \end{array}$$ (26)

The system space state equation is established as follows:


$$\begin{array}{c} \left\{ \begin{array}{c} {\dot{z}}_{1}=az_{1}+z_{2}+bu\\ {\dot{z}}_{2}=0\\ y=z_{1} \end{array}\right. \end{array}$$ (27)

Where, the state variables $z_{1}=\omega_{m}$, $z_{2}=-d$, and output $y=\omega_{m}$, taking $z_{1}$ and $z_{2}$ as observation objects, a high gain feedback of the speed estimation error e is established, and a high-gain extended disturbance observer is designed as follows:


$$\begin{array}{c} \left\{ \begin{array}{c} e=z_{1}-{\hat{z}}_{1}\\ {\dot{\hat{z}}}_{1}=a{\hat{z}}_{1}+{\hat{z}}_{2}+bu+\frac{\alpha_{1}}{\mu }e\\ {\dot{\hat{z}}}_{2}=\frac{\alpha_{2}}{\mu^{2}}e \end{array}\right. \end{array}$$ (28)

Where, $z_{1}$ is the actual input mechanical angular velocity, ${\hat{z}}_{1}$ is the estimated mechanical angular velocity of the observer, ${\hat{z}}_{2}$ is the estimated matching disturbance, μ>0, and $\alpha_{1}$ and $\alpha_{2}$ are positive real numbers. Therefore, the designed high-gain extended observer is expressed as


$$\begin{array}{c} u=\frac{1}{D}\left[\frac{l{\hat{z}}_{2}}{J}+px_{1}+q{\left|x_{1}\right|}^{\lambda }sgn\left(x_{1}\right)+\frac{B}{J}\omega_{m}+cs+|s{|}^{s\omega }f(s)\right] \end{array}$$ (29)

Where, l is the observation gain of the extended observer.

### Design of beat free predictive current controller

Selecting the motor current $i_{d},i_{q}$ as the state variable, and for a SPMSM, we rewrite Eq (1) as follows:


$$\begin{array}{c} \left\{ \begin{array}{c} \dot{i_{d}}=-\frac{Ri_{d}}{L}+\omega_{e}i_{q}+\frac{u_{d}}{L}\\ \dot{i_{q}}=-\omega_{e}i_{d}-\frac{Ri_{q}}{L}-\frac{\omega_{e}\phi_{f}}{L}+\frac{u_{q}}{L} \end{array}\right. \end{array}$$ (30)

As the sampling time $T_{s}$ is sufficiently short, the first-order Euler forward discretization strategy is used to discretize Eq (30), and the following is obtained:


$$\begin{array}{c} \left\{ \begin{array}{c} \dot{i_{d}}=\frac{i_{d}(k+1)-i_{d}(k)}{T_{s}}\\ \dot{i_{q}}=\frac{i_{q}(k+1)-i_{q}(k)}{T_{s}} \end{array}\right. \end{array}$$ (31)

Where, $T_{s}$ is the sampling time. Substituting Eq (31) into Eq (30) and writing the resulting expression in matrix form we obtain:


$$\begin{array}{c} \left[\begin{array}{c} i_{d}(k+1)\\ i_{q}(k+1) \end{array}]=[\begin{array}{cc} 1-\frac{T_{S}R}{L} & T_{s}\omega_{e}(k)\\ -T_{s}\omega_{e}(k) & 1-\frac{T_{S}R}{L} \end{array}][\begin{array}{c} i_{d}(k)\\ i_{q}(k) \end{array}\right]\\ +\left[\begin{array}{c} \frac{T_{s}}{L}\\ 0 \end{array}\begin{array}{c} 0\\ \frac{T_{s}}{L} \end{array}\right][\begin{array}{c} u_{d}(k)\\ u_{q}(k) \end{array}]-[\begin{array}{c} 0\\ \frac{T_{s}\varphi_{f}\omega_{e}(k)}{L} \end{array}] \end{array}$$ (32)

Let $I(k)=[\begin{array}{cc} i_{d}(k) & i_{q}(k) \end{array}]T$ and $F(k)=[\begin{array}{cc} u_{d}(k) & u_{q}(k) \end{array}]T$, then Eq (32) can be rewritten as:


$$\begin{array}{c} I(k+1)=CI(k)+DF(k)-E \end{array}$$ (33)

Where, $C=[\begin{array}{cc} 1-T_{s}R/L & T_{s}\omega_{e}(k)\\ -T_{s}\omega_{e}(k) & 1-T_{s}R/L \end{array}]$, $D=\left[\begin{array}{c} T_{s}/L\\ 0 \end{array}\begin{array}{c} 0\\ T_{s}/L \end{array}\right]$, $E=[\begin{array}{c} 0\\ T_{s}\phi_{f}\omega_{e}(k)/L \end{array}]$.

Let $I(k+1)=I^{*}(k$ and $I^{*}(k)=[\begin{array}{cc} i_{d}^{*}(k) & i_{q}^{*}(k) \end{array}]T$, then the control output of the no-beat current controller is


$$\begin{array}{c} F(k)=\left[I^{*}(k)-CI(k)+E\right]\cdot D^{-1} \end{array}$$ (34)

Eq (34) can be written in the following form:


$$\begin{array}{c} u_{d}(k)=\left(R-\frac{L}{T_{s}}\right)i_{d}(k)-\omega_{e}(k)Li_{q}(k)+\frac{L}{T_{s}}i_{d}^{*}(k) \end{array}$$ (35)

$$\begin{array}{c} u_{q}(k)=\left(R-\frac{L}{T_{s}}\right)i_{q}(k)+\omega_{e}(k)Li_{d}(k)+\frac{L}{T_{s}}i_{q}^{*}(k)+\omega_{e}(k)\varphi_{f} \end{array}$$ (36)

### Y-Inverter analysis and design

**Fig 3:**
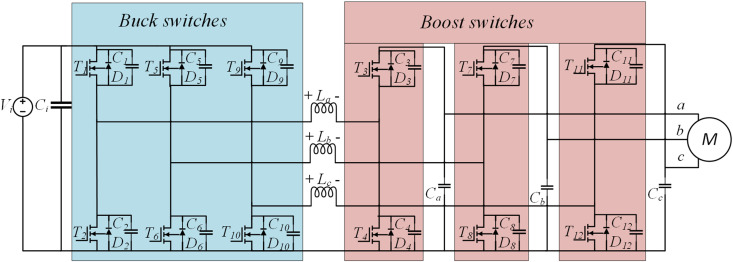
Y-Inverter topology diagram.

Y-Inverter provides four key advantages. Firstly, it can both reduce and increase pressure. Due to the step-boost characteristics of each phase module, the AC output voltage may exceed or fall below the DC input voltage. Secondly, efficiency is notable. The Y-Inverter manages transmission power *p* in a distinctive way. Typically, only three of six half-bridges are switched at any moment, ensuring a high-quality motor voltage. An integrated L-C output filter in Y-Inverters generates a continuous sinusoidal motor voltage, eliminating the need for an extra filter between the inverter and motor. Lastly, straightforward control strategies are introduced. Each phase module can be controlled independently of the other two phases in a basic configuration, akin to traditional DC/DC converters. The topology of the Y-Inverter is depicted in Fig 3.

**Fig 4:**
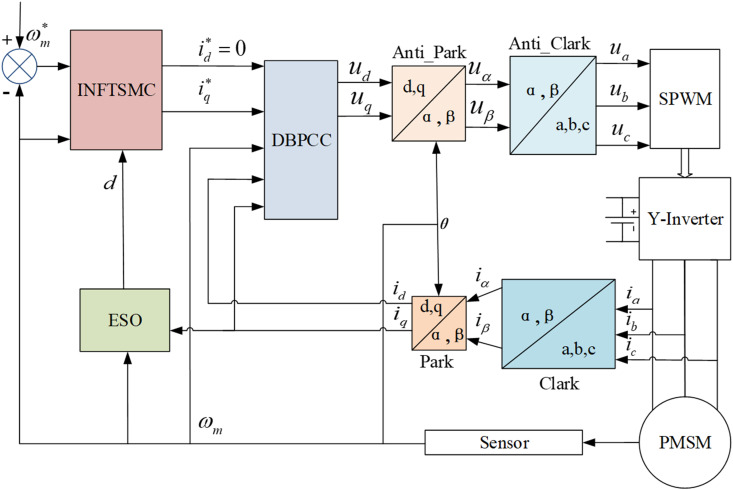
PMSM control system block diagram.

The control strategy schematic designed in this study is depicted in Fig 4.

## 3. Simulation analysis

The effectiveness and superiority of the improved non-singular fast TSMC system designed in this study were analyzed through simulations. The simulation parameters of PMSM utilized in this study are presented in Table 1.

**Table 1:** Parameters of permanent magnet synchronous motor.

Parameter	Value
Stator resistance *R*(Ω)	0.875
d-axis inductance *L*_*d*_(H)	0.0085
q-axis inductance *L*_*q*_(H)	0.0085
magnetic chain *φ*_*f*_ (Wb)	0.3
Number of poles *p*_*n*_ *p*_*n*_ *p*_*n*_	4
Inertia coefficient *J*(kg·m^-2^)	0.003
damping coefficient *B*(N·m·s)	0
DC voltage *U*(V)	311

### Operating condition 1

**Fig 5:**
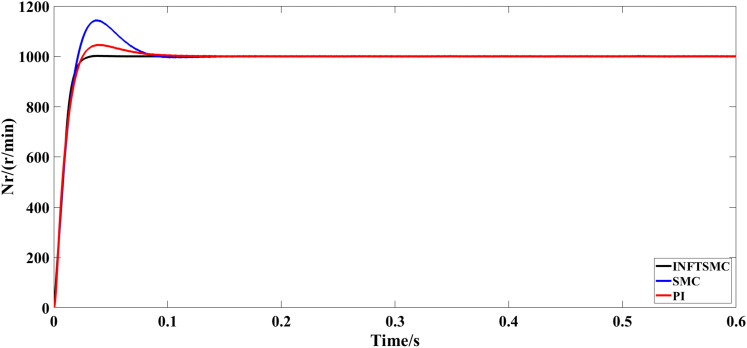
Velocity response curve(0−0.6s) under different control strategies.

**Fig 6:**
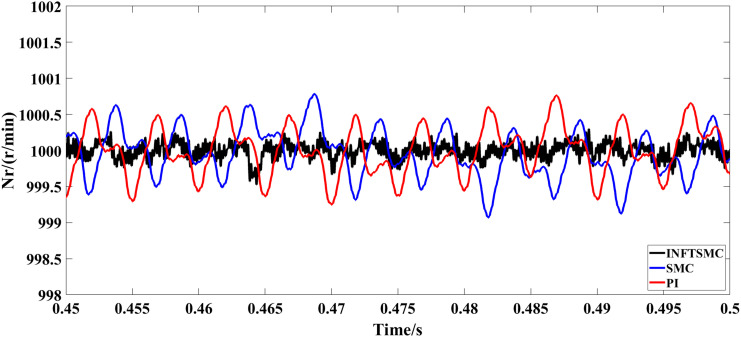
Velocity response curve(0.45−0.5s) under different control strategies.

The motor starts with no load and runs at a specified speed of 1000 r/min for 0.6 seconds. Fig 5 and Fig 6 depict the speed response curves for various control approaches. As shown in Fig 5 and Fig 6, the system speed overshoot using the traditional PI control strategy is 5%, whereas that using the traditional SMC strategy is 14.3%. The system using the new and improved non-singular fast TSMC (INFTSMC) strategy has no significant overshoot at startup and reaches steady state faster. Moreover, when the speed curve is in steady state, the speed curve shows less fluctuation, and the operation is more stable.

### Operating condition 2

**Fig 7:**
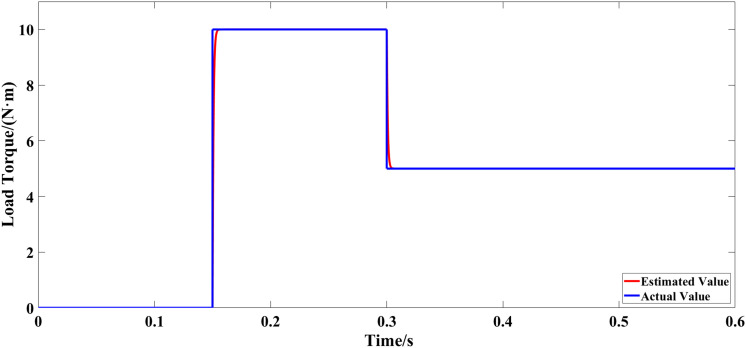
Observation curve of the observer.

**Fig 8:**
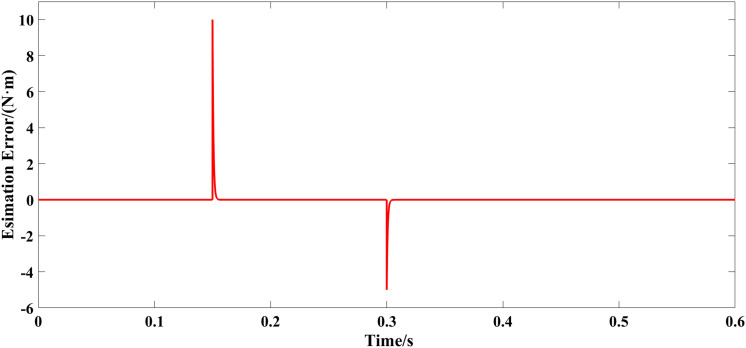
Error curve of disturbance estimation.

**Fig 9:**
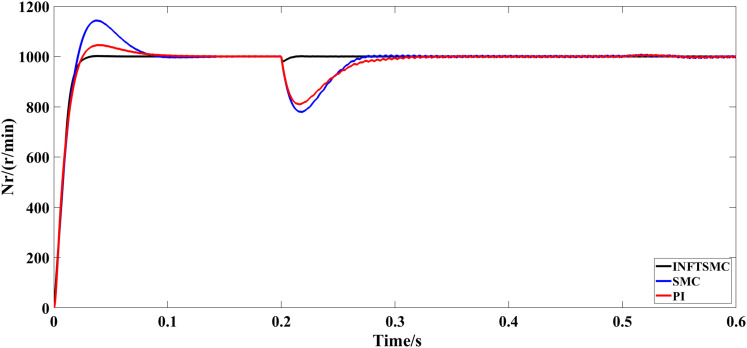
Speed waveforms(0−0.6s) of different strategies during sudden loading.

**Fig 10:**
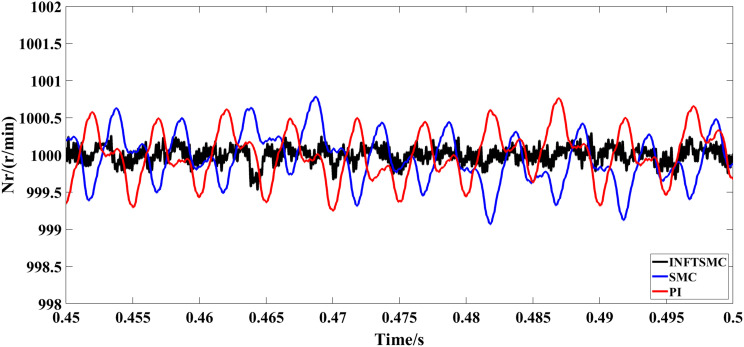
Speed waveforms(0.45−0.5s) of different strategies during sudden loading.

The motor started with no load and operated at a specified speed of 1000 r/min. When the motor is operating in a stable manner, the load torque increases abruptly to 10 N·m at 0.15 s, and decreases to 5 N·m at 0.3 s. Fig 7 displays the waveform for a specified load torque along with the observed waveform from the extended state observer. Fig 8 shows the error curve of the interference observation. Fig 9 and Fig 10 display the speed waveform under different strategies when a sudden load is applied.

From Fig 7 and Fig 8, the disturbance observer swiftly tracks system external disturbances. Under external load torque on the motor, the observer promptly traces disturbance changes. Accurate estimators compensate in the enhanced non-singular fast terminal sliding-mode controller, reducing jitter and strengthening its anti-interference ability.

Upon adopting INFTSMC, the speed decrease is only 20 r/min in Fig 9, enabling quicker restoration of stable operation and minimizing system fluctuations from external interference. As shown in Fig 9, under PI control and SMC, the system speed drops by 190 and 220 r/min respectively with external load torque on the motor. At steady state, INFTSMC systems exhibit narrower speed variations and greater stability.

**Fig 11:**
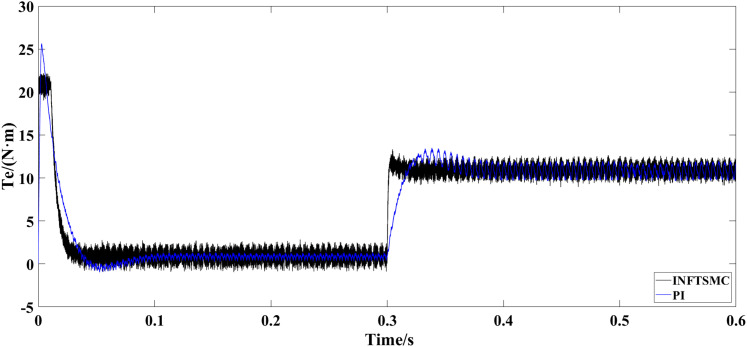
Electromagnetic torque waveform under different control strategies(INFTSMC and PI).

**Fig 12:**
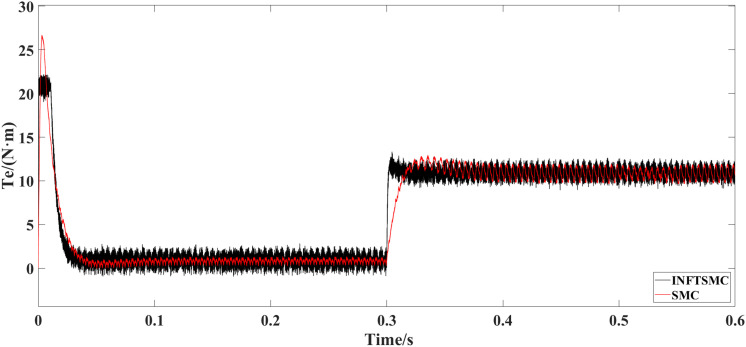
Electromagnetic torque waveform under different control strategies(INFTSMC and SMC)..

Fig 11 and Fig 12 show the electromagnetic torque waveforms under different control strategies. As can be seen from Fig 11 and Fig 12, when PI control and SMC are adopted, the maximum instantaneous torques at system startup reach 26 N·m and 27.5 N·m, respectively. Therefore, in the system utilizing INFTSMC, the maximum instantaneous value of the startup torque is relatively lower, accompanied by a smaller instantaneous value of the startup current, which is beneficial for motor protection and avoids damage caused by excessively large startup currents. In response to an increase in external load torque, after the system enters a steady state, the fluctuation of electromagnetic torque under the INFTSMC system is more gentle.

**Fig 13:**
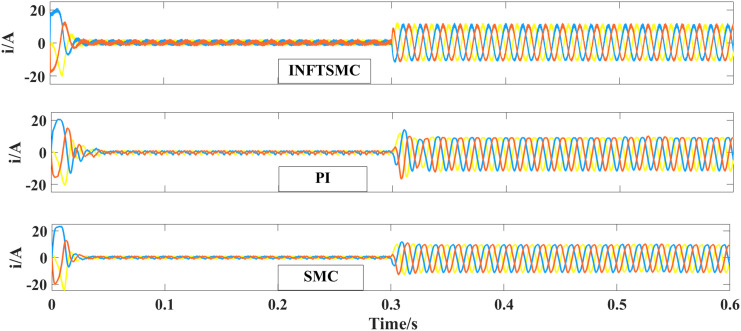
Three-phase current waveforms of different control strategies.

Fig 13 displays the waveforms of three-phase current across different control approaches. According to Fig 13, the peak system startup currents for PI control and SMC control are 24 A and 27 A, respectively. Meanwhile, the peak startup current of the INFTSMC system is 22.5 A. When subjected to external disturbances, the INFTSMC system's three-phase current stabilizes more rapidly. By examining the three-phase current, the INFTSMC strategy introduced herein offers superior dynamic and static performances for the system.

## 4. Experimental verification

Next, we present the experimental verification of the INFTSMC system using a physical experimental platform. The SPMSM parameters selected for this experiment are listed in Table 2.

**Table 2:** Parameters of permanent magnet synchronous motor.

Parameter	Value
Rated Power *P*(kW)	0.75
Rated Speed *Nr*(r/min)	3000
Rated Current *In*(A)	3
Stator resistance *R*(Ω)	0.875
d-axis inductance *L*_*d*_(H)	0.0085
q-axis inductance *L*_*q*_(H)	0.0085
magnetic chain *φ*_*f*_ (Wb)	0.3
Number of poles *p*_*n*_ *p*_*n*_ *p*_*n*_	4
Inertia coefficient *J*(kg·m^-2^)	0.003
damping coefficient *B*(N·m·s)	0
DC voltage *U*(V)	311

**Fig 14:**
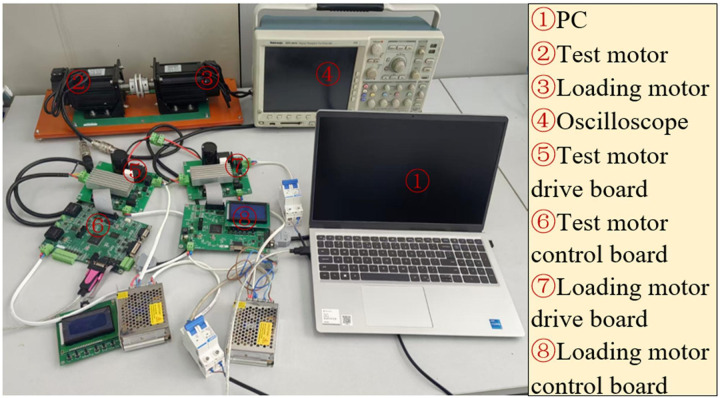
Experimental platform for control system of tabular permanent magnet synchronous motor.

The experimental platform for the SPMSM control system is illustrated in Fig 14.

The motor initiated without a load and functioned at a defined speed of 1000 r/min. Upon stable operation, the load torque abruptly rises to 10 N·m at 0.3s.

**Fig 15:**
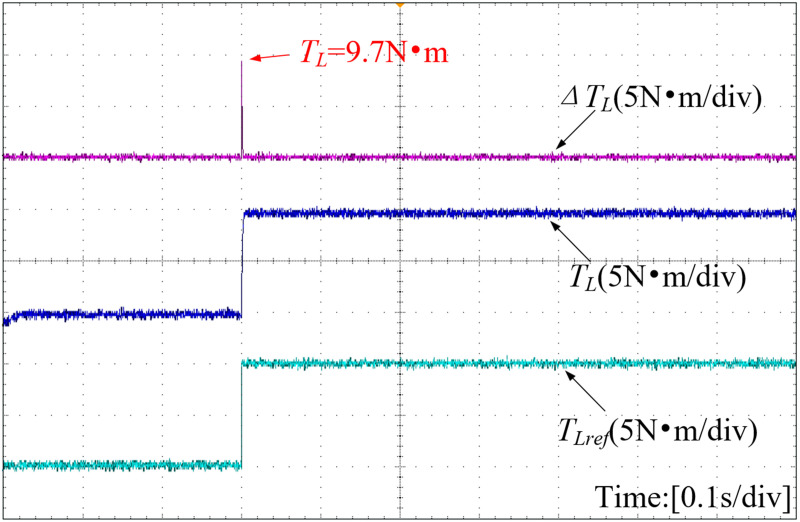
Torque and observed error curve.

Fig 15 shows that when the motor encounters external load torque, the conceived extended state observer can quickly capture the disturbance variations. Accurate disturbance estimates can be incorporated into the improved novel nonsingular fast terminal sliding mode controller to achieve the purposes of reducing chattering and enhancing anti-disturbance performance.

**Fig 16:**
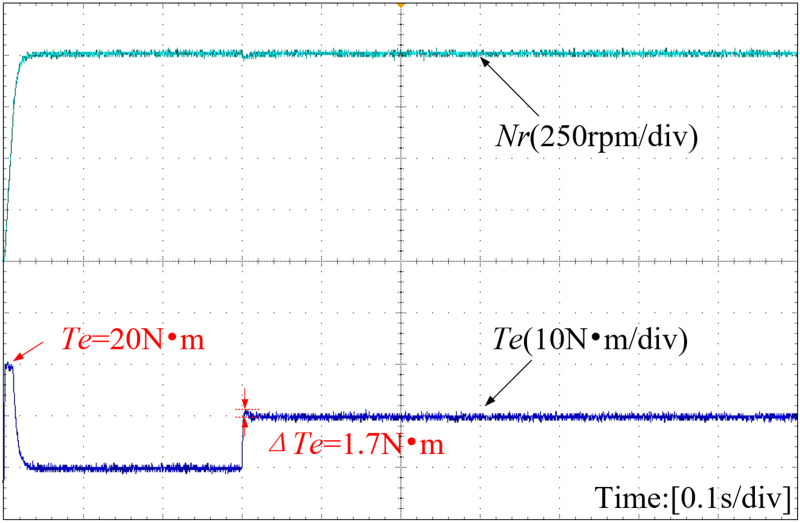
Curves of motor speed and electromagnetic torque(INFTSMC).

**Fig 17:**
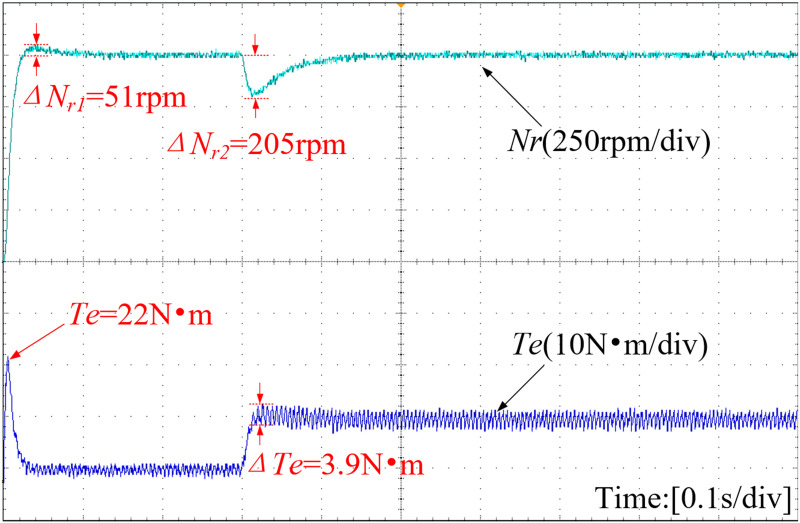
Curves of motor speed and electromagnetic torque(PI).

**Fig 18:**
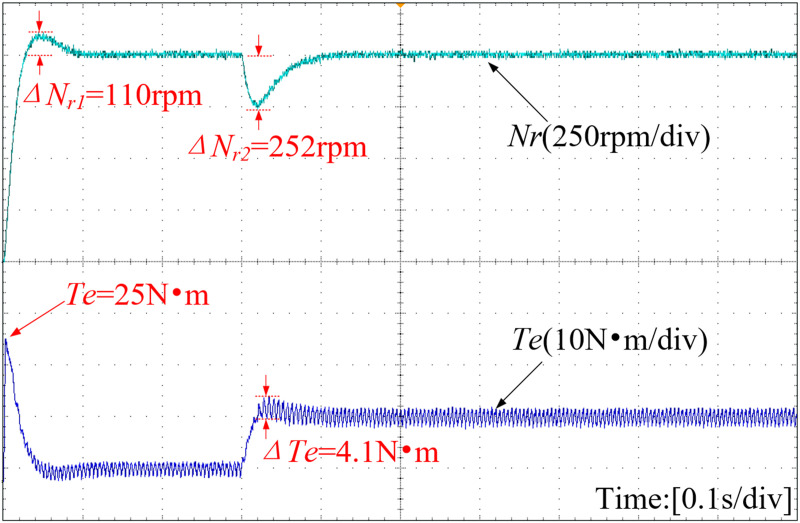
Curves of motor speed and electromagnetic torque(SMC).

Figs 16–18 present the speed curve and electromagnetic torque curve of the motor operation. With the adoption of INFTSMC in Fig 16, the system speed remains almost unchanged, enabling faster restoration of stable system operation and reducing system fluctuations caused by external disturbances. Upon a sudden load increase in Fig 17 and Fig 18, the system speed under PI control and SMC control decrease by 205 r/min and 252 r/min respectively. After reaching a steady state, the system with INFTSMC exhibits a smaller range of speed variation and operates more stably. The maximum instantaneous startup torque values for PI-controlled and SMC-controlled systems are 22 N·m and 25 N·m, respectively. Additionally, the INFTSMC system boasts the lowest maximum instantaneous startup torque at 20 N·m. Its corresponding startup current instantaneous value is also the lowest, offering superior motor protection and preventing excessive startup current damage. As external load torque rises and the system attains steady state, the INFTSMC system exhibits lesser electromagnetic torque fluctuations.

**Fig 19:**
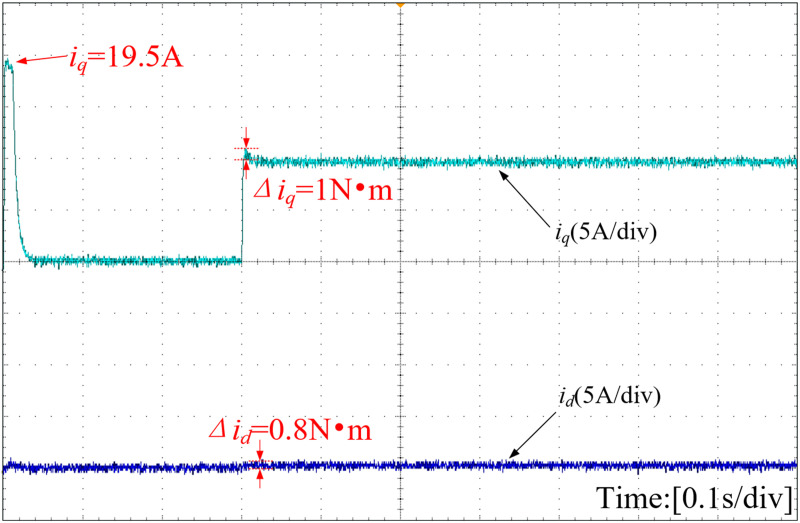
Curves of D-axis and Q-axis of the motor(INFTSMC).

**Fig 20:**
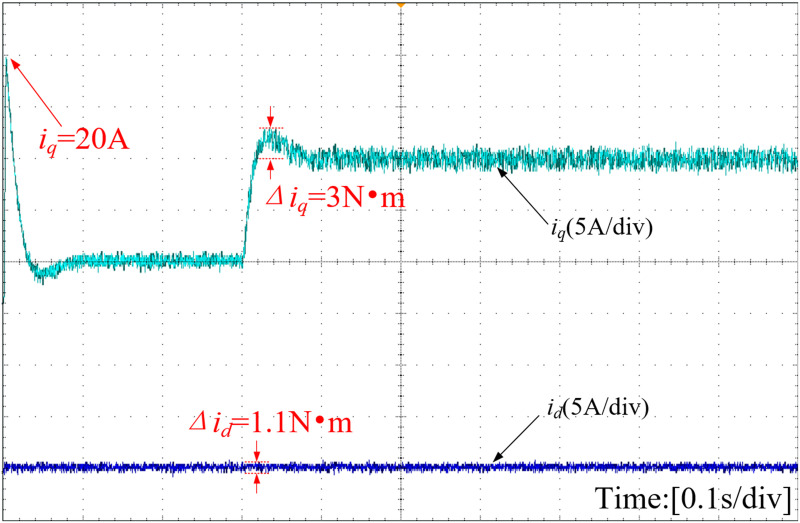
Curves of D-axis and Q-axis of the motor(PI).

**Fig 21:**
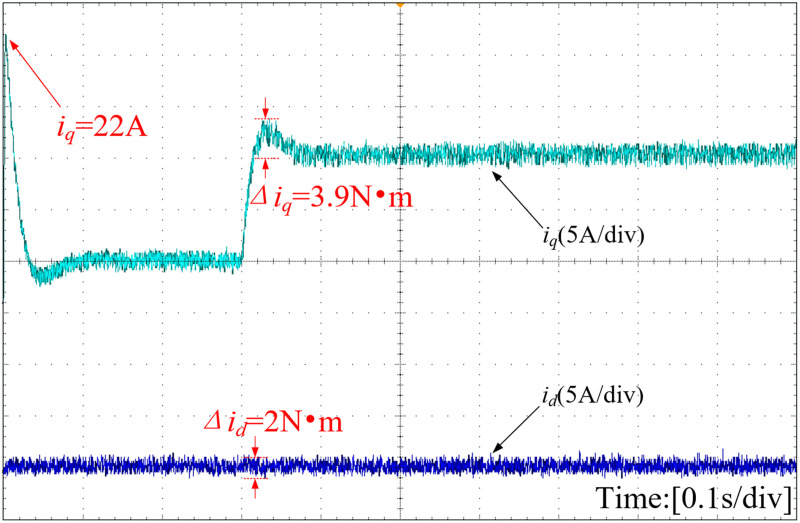
Curves of D-axis and Q-axis of the motor(SMC).

The d-axis and q-axis current waveforms, depicted under various strategies, are illustrated in Figs 19–21. The INFTSMC system boasts the lowest initial q-axis current instantaneous value at 19.5A in Fig 19. The figure reveals that the initial q-axis current instantaneous values for traditional PI control and SMC control systems are 20A and 22A in Fig 20 and Fig 21, respectively. During stable operation, the INFTSMC system displays the minimal d-axis current fluctuation. Upon encountering external disturbances, the INFTSMC system's q-axis current attains steady state faster. These findings suggest that the INFTSMC system exhibits superior robustness and enhanced dynamic and static performance.

## 5. Conclusion

The INFTSMC strategy is adopted in the velocity loop, which does not require differential state. At the same time, a high-gain extended observer is introduced to observe and compensate for the load changes of the system in real time to avoid the singular phenomenon and further enhance the control precision. The beat-free predictive current controller is adopted in the current loop, which does not introduce any adjustable parameters and can obtain better dynamic response performance. The simulation and experimental outcomes demonstrate the efficacy and advantages of this control strategy.

## References

[1] Kang EL, Yu HT, Han KW. Nonlinear gain non-singular fast terminal sliding mode control for permanent magnet synchronous motors. Electric Machines and Control. 2024;28(05):73–81.

[2] Xie ZQ, Tan X, Zhang Y. A self-tuning speed control system of PMSM based on the sliding mode observer. Journal of Hunan University of Technology. 2019;33(5):25.

[3] Tarczewski T, Grzesiak LM. Constrained State Feedback Speed Control of PMSM Based on Model Predictive Approach. IEEE Trans Ind Electron. 2016;63(6):3867–75. doi: 10.1109/tie.2015.2497302

[4] Zhang Z, Zhou YZ. An improved variable structure active disturbance rejection control for the permanent magnet synchronous motor position servo system. Chinese Journal of Scientific Instrument. 2022;43(5):263–71.

[5] Anjum W, Husain AR, Abdul Aziz J, Abbasi MA, Alqaraghuli H. Continuous dynamic sliding mode control strategy of PWM based voltage source inverter under load variations. PLoS One. 2020;15(2):e0228636. doi: 10.1371/journal.pone.0228636 32027697 PMC7004334

[6] Chen Y, Liu J, Yao ZA. PMSM Sliding Mode Control Based on Disturbance Observer and New Non-Singular Fast Terminal. Modular Machine Tool & Automatic Manufacturing Technique. 2022;(03):84–7.

[7] Qiu X, Yu L, Nan YR. A survey for control strategy of permanent magnetism linear synchronous motors. Small & Special Electrical Machines. 2005;33(10):39–43.

[8] Xu B, Zhang L, Ji W. Improved Non-Singular Fast Terminal Sliding Mode Control With Disturbance Observer for PMSM Drives. IEEE Trans Transp Electrific. 2021;7(4):2753–62. doi: 10.1109/tte.2021.3083925

[9] Hu JB, Li F, Wei GL. Theory and application of backstepping sliding mode variable structure control for uncertain systems. Systems Engineering and Electronics. 2014;36(3):519–26.

[10] Zheng JC, Wang H, Man ZH. Robust motion control of a linear motor positioner using fast nonsingular terminal sliding mode. IEEE/ASME Transactions on Mechatronics. 2015;20(4):1743–52.

[11] Yang YM, Zhu QX. Sliding mode control of permanent magnet synchronous motor based on improved extended state observer. Journal of Xi’an Polytechnic University. 2024;38(01):1–8.

[12] Labbadi M, Iqbal J, Djemai M, Boukal Y, Bouteraa Y. Robust tracking control for a quadrotor subjected to disturbances using new hyperplane-based fast Terminal Sliding Mode. PLoS One. 2023;18(4):e0283195. doi: 10.1371/journal.pone.0283195 37093830 PMC10124896

[13] Xu K. Design and optimization of the fuzzy sliding mode speed regulation system for permanent magnet synchronous motor. Machine Design and Manufacturing Engineering. 2020;49(5):42.

[14] Ali A, Khan Q, Ullah S, Waqar A, Hua L-G, Bouazzi I, et al. High gain differentiator based neuro-adaptive arbitrary order sliding mode control design for MPE of standalone wind power system. PLoS One. 2024;19(1):e0293878. doi: 10.1371/journal.pone.0293878 38236831 PMC10796038

[15] Ke XB, Yuan XF, Guo L. Permanent magnet synchronous motor control based on fuzzy integral sliding mode controller. Computer Technology and Development. 2020;30(6):197.

[16] Li S, Zhou M, Yu X. Design and Implementation of Terminal Sliding Mode Control Method for PMSM Speed Regulation System. IEEE Trans Ind Inf. 2013;9(4):1879–91. doi: 10.1109/tii.2012.2226896

[17] Lu E, Li W, Yang X, Liu Y. Anti-disturbance speed control of low-speed high-torque PMSM based on second-order non-singular terminal sliding mode load observer. ISA Trans. 2019;88:142–52. doi: 10.1016/j.isatra.2018.11.028 30563689

[18] Wu Y, Li G. Adaptive disturbance compensation finite control set optimal control for PMSM systems based on sliding mode extended state observer. Mechanical Systems and Signal Processing. 2018;98:402–14. doi: 10.1016/j.ymssp.2017.05.007

[19] Lan C-Y, Wang H, Deng X, Zhang X-F, Song H. Multi-motor position synchronization control method based on non-singular fast terminal sliding mode control. PLoS One. 2023;18(6):e0281721. doi: 10.1371/journal.pone.0281721 37319306 PMC10270599

[20] Fu DX, Zhao XM. Back stepping terminal sliding mode position control based on neural network observer. Control Theory & Applications. 2023;40(1):132–8.

[21] Yin C, Wang S, Gao J, Li X. Trajectory tracking for agricultural tractor based on improved fuzzy sliding mode control. PLoS One. 2023;18(4):e0283961. doi: 10.1371/journal.pone.0283961 37023110 PMC10079061

[22] Fang X, Wang LM, Zhang K. High order non-singular fast terminal sliding mode control of permanent magnet linear motors based on disturbance observer. Journal of Electrical Engineering. 2023;38(2):409–21.

[23] Zhao F, Luo W. Sensor hybrid control for permanent synchronous motor using fuzzy sliding mode controller and two-stage filter observer. Control Theory & Applications. 2020;37(8):1865.

[24] Jiang P, Song LY. PMSM Integral Sliding Mode Control Based on Fuzzy Disturbance Observer. Computer Applications and Software. 2020;37(1):93.

[25] Wang J, Xu D, Zhou H, Bai A, Lu W. High-performance fractional order terminal sliding mode control strategy for DC-DC Buck converter. PLoS One. 2017;12(10):e0187152. doi: 10.1371/journal.pone.0187152 29084255 PMC5662226

[26] Li Z, Zhang ZH, Wang JS, Yan ZB. Nonsingular Fast Terminal Sliding Mode Control Strategy for PMLSM Based on Disturbance Compensation. Journal of Power Electronics. 2024;24:249–57.

[27] Tang JY, Zhang B. PMLSM high-order adaptive non-singular fast terminal sliding mode control based on extended state observer. Research and Development. 2024;43(04):124–32.

[28] Zhu YH, Yu YJ. SPMSM speed sensorless control based on non-singular fast terminal fuzzy sliding mode controller. Electric Machines Control Application. 2020;47(10):17–23.

[29] Li T, Liu XD. Non-Cascade Fast Nonsingular Terminal Sliding Mode Control of Permanent Magnet Synchronous Motor Based on Disturbance Observers. Journal of Electrical Engineering & Technology. 2021;17(02):1–15.

[30] Nasim U, Bhatti AR, Farhan M, Rasool A, Butt AD. Finite-time robust speed control of synchronous reluctance motor using disturbance rejection sliding mode control with advanced reaching law. PLoS One. 2023;18(9):e0291042. doi: 10.1371/journal.pone.0291042 37695775 PMC10495029

[31] Cui H. Application of sliding mode variable structure control algorithm in PMSM vector control system in complex environment. PLoS One. 2024;19(9):e0308417. doi: 10.1371/journal.pone.0308417 39269933 PMC11398649

